# P070 Polymyalgia rheumatica; but what lay silent in the shadows?

**DOI:** 10.1093/rap/rkag113.100

**Published:** 2026-11-03

**Authors:** Syeda Afshan Tabassum, Shumaila Baloch, Karen Douglas

**Affiliations:** The Dudley Group NHS Foundation Trust, Dudley, United Kingdom; The Dudley Group NHS Foundation Trust, Dudley, United Kingdom; The Dudley Group NHS Foundation Trust, Dudley, United Kingdom

## Abstract

**Introduction:**

Polymyalgia rheumatica (PMR) is a common inflammatory rheumatic disease in older adults and is typically characterised by proximal girdle stiffness and pain, elevated inflammatory markers and a rapid response to glucocorticoids. Although most patients respond well to corticosteroids, atypical clinical features, persistent inflammatory markers, prolonged steroid dependence or recurrent relapses should warrant a diagnostic re-evaluation. We report a case series of three patients initially diagnosed, by a rheumatology consultant, with PMR whose subsequent clinical course revealed a diagnosis of axial spondyloarthritis (axSpA).

**Case description:**

We report 3 women in their late 70s who had previously been diagnosed with PMR based on typical clinical features, including shoulder and hip girdle pain, prolonged early morning stiffness, elevated inflammatory markers and an initial response to prednisolone. All three had repeated relapses, difficulty tapering corticosteroids, and fluctuating inflammatory markers.

Patient 1 underwent extensive investigation for giant cell arteritis, malignancy and large-vessel vasculitis, all of which were unremarkable.

Patient 2, 20 years after the initial diagnosis presented acutely with severe thoracic pain and markedly elevated inflammatory markers, prompting an MRI to exclude discitis.

In both patients 1&2 MRI demonstrated characteristic features of axSpA, including fused sacroiliac joints.

Patient 3 underwent Xray and CT, which demonstrated cervical syndesmophytes, fused sacroiliac joints and aortic root involvement, consistent with longstanding axSpA.

Reassessment identified previously unrecognised chronic back pain in all 3 patients. Patient 3 had remained on corticosteroids for more than 30 years, developing significant glucocorticoid-related complications. Following revision of the diagnosis to axSpA, patient 1 commenced adalimumab with subsequent clinical improvement.

**Discussion:**

PMR remains a significant diagnostic challenge because its characteristic phenotype represents a common inflammatory presentation shared by several rheumatic diseases. While most patients demonstrate a rapid response to low-dose glucocorticoids, atypical features, persistent inflammatory activity, recurrent relapses, or prolonged steroid dependence should prompt reconsideration of the diagnosis. Our case series illustrates how axSpA may masquerade as PMR for years before characteristic radiographic and clinical features become apparent. Furthermore, diagnostic bias contributes to delayed recognition of axSpA in women. Historically regarded as a predominantly young male disease, women and patients >40years old often present atypically and experience longer diagnostic delays, increasing the risk of misdiagnosis.

The overlap between PMR and its mimics is underpinned by shared immunological pathways. IL-6 is central to PMR pathogenesis, resulting in constitutional symptoms and elevated acute-phase reactants. Similar IL-6–driven inflammation occurs in large-vessel vasculitis and late-onset rheumatoid arthritis, producing a comparable early presentation. IL-6 also promotes differentiation of naïve CD4+ T cells into Th17 cells, increasing IL-17A production. This Th17/IL-17A axis is a key pathogenic mechanism in axSpA. Enthesitis involving the shoulder and pelvic girdles in late onset axSpA may closely mimic the bursitic pain distribution of PMR, creating a substantial diagnostic pitfall.

Shared inflammatory pathways also explain the transient responsiveness of PMR mimics to glucocorticoids, often delaying recognition of the underlying disease. Conventional management relies on corticosteroids, with methotrexate used as a steroid-sparing agent in selected patients. Recent evidence from the Phase III REPLENISH trial demonstrated that secukinumab improved sustained remission and reduced cumulative glucocorticoid exposure in relapsing PMR. Similarly, tocilizumab has shown efficacy in refractory disease. These advances highlight the importance of accurate diagnosis and timely recognition of PMR mimics to facilitate mechanism-based, steroid-sparing treatment.

**Key learning points:**

• PMR is a common inflammatory disease of older adults, but several conditions can closely mimic its presentation, particularly axSpA, large-vessel vasculitis, and late-onset rheumatoid arthritis.

• A rapid response to low-dose glucocorticoids supports, but does not confirm, a diagnosis of PMR, as many inflammatory mimics demonstrate a similar initial improvement.

• Persistent elevation of inflammatory markers, recurrent relapses during steroid tapering, prolonged steroid dependence, atypical age of onset, or inflammatory spinal symptoms should prompt diagnostic re-evaluation.

• Late-onset axSpA may present predominantly with shoulder and hip girdle pain and stiffness, closely resembling PMR and resulting in significant diagnostic delay.

• Recognise that gender bias in axSpA diagnosis may contribute to delayed identification in women, who can present with atypical features and may be misclassified as having alternative inflammatory conditions such as PMR.

• Advanced imaging, particularly MRI of the spine and sacroiliac joints, can identify inflammatory changes suggestive of axSpA when the clinical course is atypical.

• Delayed recognition of PMR mimics may lead to prolonged glucocorticoid exposure and associated complications, including osteoporosis, fragility fractures, cataracts, and recurrent infections.

• Shared IL-6 and Th17/IL-17A inflammatory pathways help explain the clinical overlap between PMR and several of its mimics.

• Emerging targeted therapies, including IL-17A inhibition and IL-6 receptor blockade, are expanding treatment options and supporting a more steroid-sparing approach to disease management.

• Regular clinical reassessment is essential in patients whose disease course deviates from the expected natural history of PMR.

• Women with axial spondyloarthritis may underreport inflammatory back pain, resulting in delayed diagnosis. Careful assessment for inflammatory back pain is essential in atypical or treatment-resistant PMR presentations.

**P070 Table 1:** Patient characteristics

Characteristics	Patient 1	Patient 2	Patient 3
Age at presentation of PMR	67	57	46
CRP at presentation	109	20	35
Post steroid CRP	22	11	22
Imaging results	*SIJ XR:* SI joints appear reasonably normal *MRI spine:* Inflammatory changes consistent with active spinal enthesitis and bilateral sacroiliitis	*SIJ XR: *SI joints appear irregular – longstanding changes *MRI spine:* Multilevel inflammatory spinal changes with fatty lesions, suspicious for inflammatory arthropathy *CT scan:* Partial sacroiliac fusion, thoracic ankylosis, consistent with inflammatory arthropathy	*SIJ XR:* There was fusion of the sacroiliac joints *CT scan:* Bilateral sacroiliac fusion with widespread inflammatory and degenerative spinal changes
HLA B27	Unknown	Positive	Positive
Final diagnosis	AxSpA	AxSpA	AxSpA
**Timeline to final diagnosis**	**4 years**	**22 years**	**30 years**

